# Case report and literature review: the effect of surgical management in intrathoracic pseudomyxoma peritonei

**DOI:** 10.3389/fsurg.2026.1779316

**Published:** 2026-05-18

**Authors:** De-Wang Ren, Zhi-Yuan Wang, Li-Bin Miao, Wei-Qiang Lu, Run-Yang Ma, Xue-Jun Dou

**Affiliations:** Department of Thoracic Surgery, Aerospace Center Hospital, Beijing, China

**Keywords:** cytoreductive surgery, diagnosis, hyperthermic chemotherapy, intrathoracic pseudomyxoma peritonei, prognosis

## Abstract

**Background:**

Intrathoracic metastasis in patients with pseudomyxoma peritonei is extremely rare and carries a poor prognosis. No standard treatment recommendations have been established, and it remains unclear whether surgical treatment can improve the long-term prognostic outcomes.

**Cases description:**

A retrospective analysis of the clinical findings of six intrathoracic pseudomyxoma peritonei patients that underwent surgical treatment at the Aerospace Center Hospital between March 2013 and June 2022 was conducted. Five patients exhibited evidence of direct extension into the pleural cavity, and four underwent parietal/visceral pleurectomy to remove as much of the mucinous tissue from the thoracic cavity as possible. One patient underwent the resection of disseminated pleural and lung lesions followed by hyperthermic intrathoracic chemotherapy (HITHOC). One patient exhibited pulmonary metastases and underwent lobectomy. Macroscopic tumor resection was performed in all patients. Patients were monitored for a mean follow-up time of 24 months, and exhibited 1- and 3-year survival rates of 100% (6/6) and 33.3% (2/6), respectively.

**Conclusion:**

Intrathoracic pseudomyxoma peritonei is a rare clinical disease associated with a poor prognosis that primarily results following injury to the diaphragm during the surgical treatment of pseudomyxoma peritonei. Cytoreductive surgery to treat thoracic tumors in combination with HITHOC can alleviate the symptoms of this condition and can contribute to better prognostic outcomes. However, it is important to remain attentive to the potential for perioperative complications.

## 1 Introduction

Pseudomyxoma peritonei (PMP) is a rare disease affecting just 3–4/1,000,000 persons per year (1). PMP patients exhibit diffuse intra-abdominal mucous-like material together with large numbers of mucinous lesions on the omentum and peritoneal surfaces (1, 2). The appendix is often the site of the primary lesion in affected patients, with low-grade lesions often coinciding with the presence of low-grade mucinous neoplasms of the appendix. In contrast, high-grade lesions are generally related to the development of mucinous adenocarcinomas. In some cases these peritoneal mucinous tumors can also originate from other sites including the ovaries, mesenteric cysts, or colorectal tissue (3–7). While PMP can develop across a wide age spectrum, it is most common in middle-aged or older individuals, and affects females more often than males (8). Clinical symptoms in affected patients include transient fevers, mucinous ascites, persistent abdominal pain, abdominal masses, and symptoms consistent with an incomplete intestinal obstruction. During the later stages of the disease patients may experience weight loss, a loss of appetite, and anemia. Cytoreductive surgery (CRS) in combination with hyperthermic intraperitoneal chemotherapy (HIPEC) is generally considered to be the most effective treatment for PMP, leading to the significant prolongation of patient survival (9, 10).

In extremely rare cases, PMP can spread to the thoracic cavity (11). Such cases of intrathoracic PMP are associated with poor prognostic outcomes, and no standard treatment recommendations have been established for affected patients such that systemic palliative chemotherapy is often performed in an effort to prolong patient survival (12). Currently, data regarding the long-term prognostic outcomes associated with the surgical treatment of intrathoracic PMP are lacking, as there have been very few published case reports discussing the resection of intrathoracic lesions in affected individuals. In this study, we retrospectively present the clinical data from 6 PMP patients with intrathoracic metastases that underwent treatment in our department from 2013 to 2022 and discuss the related literature to improve clinical awareness and understanding of this very rare disease.

## 2 Case series

**Figure 1:**
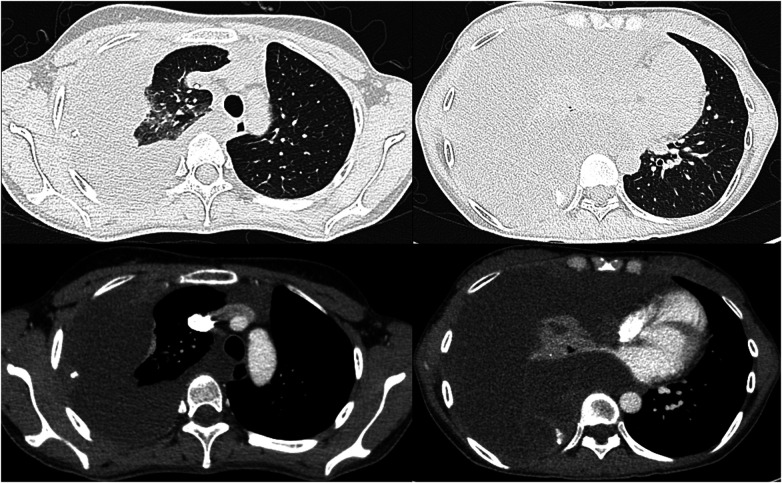
CT shows a large amount of relatively homogeneous fluid-density shadow in the right thoracic cavity, with multiple cystic lesions and patchy soft tissue-density shadows within.

Clinical data from 6 intrathoracic PMP patients that underwent surgical treatment at our hospital between March 2013 and June 2022 were retrospectively reviewed (Table 1). The inclusion criteria for CRS/HITHOC include sufficient cardiopulmonary reserve, technically resectable intrathoracic lesions, and improvement of symptoms. All 6 patients (3 male, 3 female) were diagnosed with low-grade PMP. The primary complaints in these patients included dyspnea, shortness of breath, and chest tightness. Chest computed tomography (CT) scans revealed extensive pleural effusion and compressive atelectasis or metastatic lung tumors (Figure 1). The medical history for these patients ranged from 1 to 26 months (average: 15 months), and all 6 patients exhibited a history of undergoing abdominal surgery, including 5 that had undergone multiple rounds of CRS combined with HIPEC for PMP and one that had undergone an appendectomy 20 years previously to treat acute appendicitis. The mean interval between abdominal CRS and thoracic surgery was 58.2 months (range: 10–144 months).

**Table 1:** The clinical characteristics of patients.

Patient	Age	Treatment time	Pattern of extension	Treatments	Present status	Follow-up period	The type of recurrence
Case1	63	March 2013	PM	Lobectomy	Died with disease	19 months	Abdominal cavity
Case2	58	February 2017	Dis	CRS+ HITHOC Pleurectomy Wedge resection	Died with disease	47 months	Abdominal cavity
Case3	46	July 2019	Dis	CRS Pleurectomy	Died with disease	20 months	Abdominal cavity and thoracic cavity
Case4	53	July 2020	Dis	CRS Pleurectomy	Died with disease	12 months	Thoracic cavity
Case5	32	March 2021	Dis	CRS Pleurectomy	Disease recurrence	33 months	Abdominal cavity
Case6	50	June 2022	Dis	CRS Pleurectomy	Died with disease	13 months	Abdominal cavity

PM, pulmonary metastasis; Dis, dissemination; CRS, cytoreductive surgery; HITHOC, hyperthermic intrathoracic chemotherapy.

**Figure 2:**
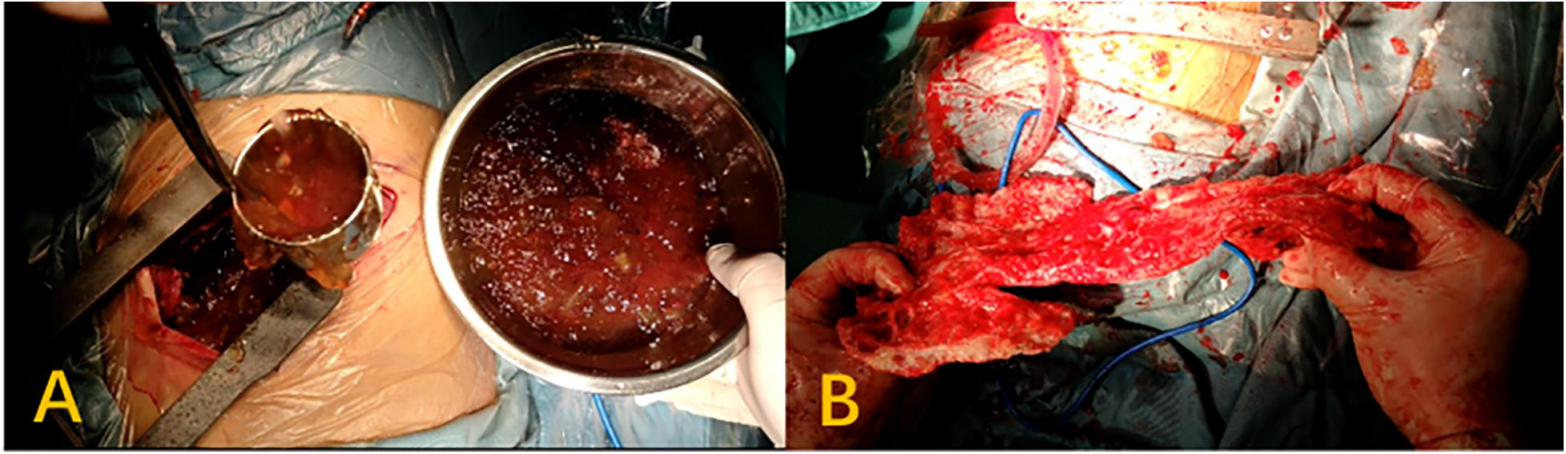
**(A)** Jelly-like pleural effusion. **(B)** Significant thickening of the visceral pleura.

All patients in this case series had undergone CRS to resect all visible intrathoracic mucinous lesions (Figure 2). When lesions were determined to be the result of lung invasion or pulmonary metastasis, the lesions were resected along with surrounding tissue. In patients exhibiting pleural dissemination, all visible tumors were removed through a combination of pleurectomy, simple lesion removal, diaphragm resection, or lung and tumor resection. After disseminated lesions had been resected, one patient was administered HITHOC using the following protocol: 2000–3000 mL of saline containing 80 mg of cisplatin (at 42.5–43.5 °C) was administered into the thoracic cavity. Two intrathoracic drains (1 inflow drain, 1 outflow grain) were positioned and connected to a hyperthermia chemotherapy perfusion machine (Jilin Minda Company products, RHL-2000B). This solution was circulated for ∼1 h following lesion resection.

Thoracic CRS for disseminated disease or lung resection for pulmonary metastases was performed in all 6 patients in this study cohort. Of the 5 patients exhibiting disseminated disease, 4 underwent parietal/visceral pleurectomy and the removal of the mucus in the thoracic cavity to the greatest extent possible, while 1 patient underwent the resection of all disseminated pleural and lung lesions followed by HITHOC. One patient exhibited pulmonary metastases and was treated via lobectomy.

**Figure 3:**
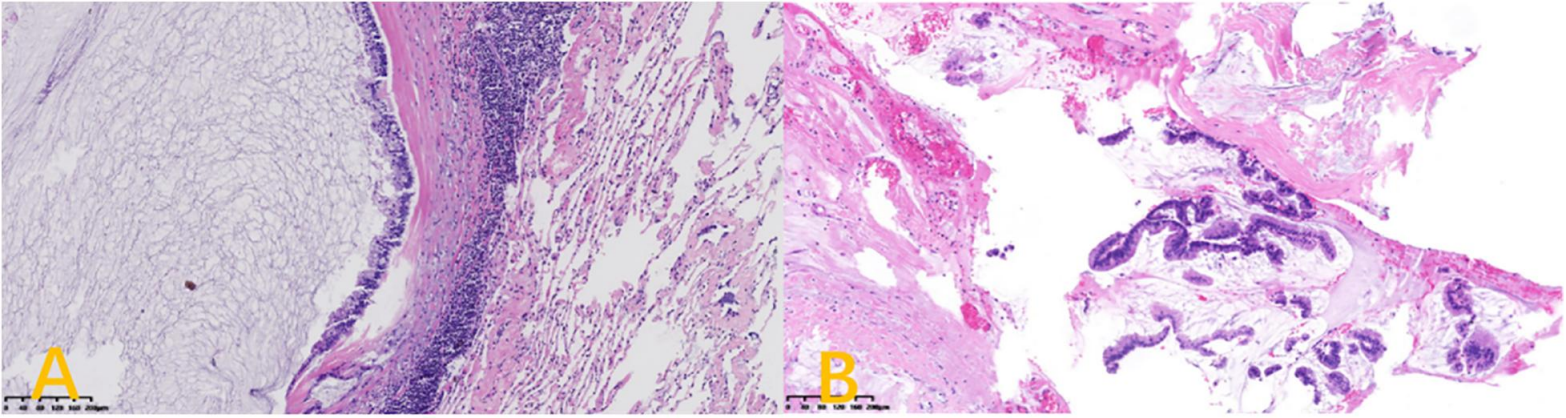
H&E staining of lung **(A)** and pleural **(B)** tissue specimens: low-grade cytologic atypia and tumor invasion (100×magnification).

Postoperative histopathology results in all 6 cases were consistent with a diagnosis of low-grade peritoneal pseudomyxoma derived from the appendix (Figure 3). The pathological report suggests the formation of mucus lakes and mild atypical glandular infiltration in the fibrous adipose tissue submitted for examination. Combined with clinical history, it is consistent with the involvement of low-grade peritoneal pseudomyxoma. One of these patients developed incomplete intestinal obstruction on day 5 after surgery, but was discharged following symptomatic treatment. There were no instances of operative death or serious postoperative complications in these patients. The average hospital length of stay for these patients is 16 days. Patients underwent follow-up for up to 47 months (until January 2024). The most common causes of mortality were thoracic or abdominal tumor recurrence and respiratory failure.

## 3 Discussion

PMP is a rare malignancy of the peritoneum. Primary PMP lesions are most commonly derived from the appendix, and in its advanced stages the disease can often result in intestinal obstruction and consequent mortality. Treatment efforts for affected patients generally center around a combination of CRS and HIPEC, leading to improved 5-year survival rates of 69%–75% and 10-year survival rates that reportedly rise from 30% to 57% with treatment (8–10). The success of these treatment strategies and the associated prolongation of patient survival have also led to the detection of previously overlooked mucinous tumor progression within the pleural cavity. PMP tumor distributions generally follow the patterns of peritoneal fluid movement and absorption such that they tend to accumulate on the omentum and subdiaphragmatic surfaces. Metastatic progression typically results from direct spreading within the abdominal cavity, whereas there have been only rare reports of lymphatic and hematogenous metastases (8). Extra-abdominal PMP thus remains a rare but well-documented condition, with the pleural cavity being the most common site of such extra-abdominal progression.

### 3.1 Mechanism of metastases

Intrathoracic PMP metastases primarily present in the form of lung metastases and pleural effusion, and the morphological features of these lesions are poorly understood. The interval between initial PMP diagnosis and the detection of thoracic metastasis can range from 1 to 15 years (12). Mechanisms previously reported to facilitate such metastasis include direct diaphragmatic invasion, acquired or congenital pleuroperitoneal communication, or intraoperative damage to the diaphragm in patients undergoing peritonectomy (13). Postoperative rates of PMP recurrence are high. Effective treatment of PMP patients often entails multiple rounds of abdominal tumor debulking in order to eliminate mucus and tumor tissues from the abdominal cavity, and these repeated CRS procedures, particularly when performing subdiaphragmatic peritoneal resection, have the potential to cause iatrogenic damage to the diaphragm. PMP thoracic extension primarily results following such intraoperative diaphragmatic damage during abdominal tumor debulking surgery, with previously reported incidence rates of up to 75% (14). As such, this extension is thought to be the result of dissemination rather than metastasis. Thoracic extension at the time of initial PMP diagnosis, in contrast, is very rare (15).

In a study of 426 PMP patients, Pestieau et al. (16) found that 23 (5.4%) developed thoracic metastases. Of these cases, 12 resulted from iatrogenic injuries to the diaphragm, with 6 patients having experienced minor diaphragm penetration while undergoing subdiaphragmatic peritonectomy that was immediately repaired via suturing, while the remaining 6 underwent central hemidiaphragm tendon resection as a consequence of subdiaphragmatic tumor invasion. Four of these cases exhibited pleural involvement when initially diagnosed with PMP, while 7 developed pleural PMP during follow-up despite any recorded penetration during peritoneal cytoreduction procedures and the presumed integrity of the diaphragm. As such, the mechanisms underlying metastatic progression in these patients remain uncertain and may be the result of minor unrecognized diaphragmatic damage during surgical treatment. One retrospective analysis of 626 PM patients reported intrathoracic metastases in 42 patients (6.7%), including 22 with pulmonary metastases, 10 with intrathoracic metastases, and 10 with both (17). In the present study, five of the included patients underwent multiple rounds of CRS combined with HIPEC to treat PMP such that iatrogenic damage to the diaphragm is the most likely cause of thoracic metastasis in these cases. The sixth patient underwent appendectomy 20 years ago due to acute appendicitis and this procedure did not result in damage to the diaphragm. However, a review of the imaging findings from this patient was consistent with pulmonary metastasis.

### 3.2 Treatment and prognosis

As data from multicenter studies and large randomized clinical trials focused on the treatment of intrathoracic PMP metastases are lacking, the optimal treatment of this rare disease remains a serious clinical challenge. No standard treatment regimen has been developed to date, and affected patients face high rates of morbidity and mortality such that palliative care is recommended in most cases. The establishment of a standardized therapeutic regimen is vital to prolonging patient survival. Kitai (18) proposed the resection of metastatic thoracic lesions when CRS and HIPEC were able to effectively control abdominal lesions, and successfully treated patients tend to exhibit a good prognosis and survival outcomes. Some specialized centers have thus adopted a combination of CRS and intraoperative hyperthermic intrathoracic chemotherapy (HITHOC) to treat intrathoracic PMP metastases. HITHOC is an intraoperative and topical administration of chemotherapeutic drugs with simultaneous warming of the thoracic cavity. Chemotherapy and hyperthermia have a synergistic effect in determining the increased local cytotoxicity of tumor cells (19). Therefore, HITHOC allows better loco-regional tumor control, enhancing surgery treatment and improving disease-free and overall survival of patients with pleural malignancies (20, 21). Kawaguchi et al. (13), for example, combined thoracic CRS with HITHOC to treat dissemination or lung resection to treat pulmonary metastases in a cohort of 17 patients. Their patient population achieved a 46.1% 5-year overall survival rate and a 34.9% relapse-free survival rate, with a median survival duration of 45.5 months and survival of up to 93.9 months. In a separate analysis of 23 patients with intrathoracic PMP metastases, Pestieau et al. (16) reported that 8 patients underwent thoracic CRS combined with HITHOC while 4 only underwent palliative surgery, with a median survival interval of 55 months. One recent retrospective study (22) of 64 patients with intrathoracic PMP metastases, of whom 20 received CRS/HITHOC. The HITHOC group demonstrated a trend toward improved OS after pleural recurrence compared with the non-HITHOC group (median 58.5 vs. 16.4 months; *p* = 0.063). There have also been some case reports describing the surgical management of intrathoracic lesions following abdominal CRS (Table 2) (12, 18, 23–26).

**Table 2:** Previous case reports on treatments for intrathoracic PMP.

Report	Number of cases	Pattern of extension	Treatments	Present status	Follow-up period
Kitai (18)	1	PM	Wedge resection	Died with disease	1 year
Mortman (23)	3	PM	2: Lobectomy 1: Wedge resection	No evidence of disease No evidence of disease	2 years 8 years
Geisinger (24)	2	PM	1: Lung resection 1: Wedge resection	No evidence of disease Not discribed	2 years Not discribed
Khan (26)	1	PM	Bilateral staged metastatectomies	No evidence of disease	9 months
Ababneh (12)	1	Dis	CRS+ HITHOC	Complete response	11 months
Senthil (25)	1	Dis	CRS+ HITHOC	No evidence of disease	6 months

PM, pulmonary metasis; Dis, dissemination; CRS, cytoreductive surgery; HITHOC, hyperthermic intrathoracic chemotherapy.

In the present case series, four patients underwent parietal/visceral pleurectomy and the removal of mucus from the thoracic cavity, while one patient underwent the resection of all disseminated pleural and lung lesions followed by HITHOC, and one patient with pulmonary metastases underwent lobectomy. These patients experienced significant improvements in their quality of life resulting from the relief of symptoms including shortness of breath, dyspnea, and chest tightness. The 1- and 3-year survival rates in this patient cohort were 100% and 33.3%, respectively. The median survival duration of patients was 19.5 months, with a minimum of 12 months and a maximum of 47 months. The patient that underwent intrathoracic CRS and HITHOC survived 47 months, which was notably longer than the survival of other patients. The most common causes of death were thoracic and abdominal tumor recurrence and respiratory failure.

### 3.3 Perioperative complications

Efforts to treat intrathoracic PMP are associated with high rates of morbidity and mortality such that intensive post-surgical observation is critical for treated patients. Kawaguchi et al. (13) reported a high rate of morbidity affecting 5 of their studied patients (29%), with 3 exhibiting empyema (18%), 1 suffering from a duodenal ulcer (6%), and 1 experiencing loss of appetite necessitating total parenteral nutrition (6%). The perioperative mortality rate in their study was 5.9%, with 1 patient having died due to empyema after pneumonectomy. Thoracic surgery may be associated with a high long-term risk of poor nutritional status, particularly for patients undergoing highly invasive thoracic procedures. In our study cohort, one patient developed an incomplete intestinal obstruction after surgery that was ultimately improved with time such that they were discharged following treatment. None of the included patients experienced perioperative mortality. HITHOC can result in complications including empyema, thrombocytopenia, bleeding, and air leak (27). In one prior study, a combination of CRS and cisplatin-based HITHOC was associated with high (up to 50%) rates of acute kidney injury (AKI), particularly for patients undergoing extrapleural pneumonectomy (28). This incidence of AKI and associated mortality may be attributable to both the characteristics of the surgical procedure and to cisplatin-related toxicity, but reports of renal toxicity are rare given that appropriate perioperative fluid management and cytoprotection can effectively preserve renal function (29, 30). Indeed, no instances of AKI were observed in this case series, potentially owing to the small number of included cases. In almost all study, Cisplatin appeared as a safe therapeutic option for intrapleural perfusion, even if there is still no consensus about the optimal dosage (11).

### 3.4 Prospect

Targeted therapy has been a research hotspot in recent years. Martinez-Quintanilla et al. (31) demonstrated through organoid and allogeneic implantation models that systemic targeted therapy can effectively control PMP tumors, and inhibition of the BRAF signaling pathway provides a new treatment opportunity for BRAF^V600E^ PMP patients with poor prognosis. Sun et al. (32) have shown that BromAc is a novel mucolytic agent composed of bromelain and acetylcysteine, which can be used to treat recurrent PMP. The targeted delivery of BromAc directly into a mucinous tumor via the transhepatic route, and post-treatment imaging revealed a significant 40% reduction in tumor burden, but further research and clinical trials are needed to validate this new method. Due to the rarity of cases, intrathoracic PMP shares similarities with the management principles of the abdominal cavity. The future treatment model will rely more on multidisciplinary diagnosis and treatment, providing comprehensive and all-round treatment plans through close cooperation among surgery, internal medicine, radiotherapy, imaging and other disciplines to achieve the best treatment effect for such patients.

## 4 Conclusion

Intrathoracic PMP is a rare clinical condition associated with poor patient outcomes that result primarily from injury to the diaphragm during surgical treatment of PMP. As such, it is essential that injury to the diaphragm be minimized wherever possible when performing surgery to treat PMP patients. The primary treatment approach available for intrathoracic PMP, given the absence of any large-scale clinical studies of this condition, entails the cytoreductive surgical treatment of thoracic tumors in combination with HITHOC. This therapeutic strategy can alleviate patient symptoms and is expected to contribute to better prognostic outcomes. However, it is critical that clinicians remain alert to the risk of severe complications during the perioperative period.

This study provides a clinical reference for patients with intrathoracic PMP, but the number of included cases is limited, and the research results need to be further validated by future multicenter prospective studies.

## Data Availability

The original contributions presented in the study are included in the article/Supplementary Material, further inquiries can be directed to the corresponding author.
